# Prospective evaluation of pin site infections in 39 patients treated with external ring fixation

**DOI:** 10.5194/jbji-6-135-2021

**Published:** 2021-04-07

**Authors:** Mats Bue, Arnar Óskar Bjarnason, Jan Duedal Rölfing, Karina Larsen, Juozas Petruskevicius

**Affiliations:** 1 Department of Orthopaedic Reconstruction, Aarhus University Hospital, Palle Juul-Jensens Boulevard 99, 8200 Aarhus N, Denmark; 2 Department of Clinical Medicine, Aarhus University, Palle Juul-Jensens Boulevard 82, 8200 Aarhus N, Denmark

## Abstract

**Introduction**: Pin site infection is a common complication to external ring fixation. While the aetiology is well described, monitoring of onset, location, and the distribution of infection among the pin sites still needs further attention. The present pilot study evaluates the feasibility of a prospective registration procedure for reporting, evaluating, and monitoring of pin site infections in patients treated with external ring fixation. This may promote communication between team members and assist decision-making regarding treatment. **Methods**: A total of 39 trauma, limb deformity, and bone infection patients (15 female, 24 males; mean age 49 years (range: 12–88)) treated with external ring fixation were followed in the outpatient clinic using the pin site registration tool. Pin site infection (Checketts and Otterburn (CO) grade, onset, location), use of oral or intravenous antibiotics, and any unplanned procedures due to pin sites complications (wire removal and/or replacement, premature frame removal, amputation, etc.) were registered until frame removal. **Results**: The mean (SD) frame time was 164 (83) d (range: 44–499). We performed 3296 observations of 568 pin sites. Pin infection was registered in 171 of the 568 pin sites (30 %), of which 112 (65 %) were categorized as CO 1, 42 (25 %) as CO 2, 9 (5 %) as CO 3, and 8 (5 %) as CO 5. Neither CO 4 nor CO 6 was observed. A total of 35 patients (90 %) encountered CO 1–3 at least once during the observation time, while 1 patient (2.5 %) developed a major infection at eight pin sites (CO 5). Antibiotics were administered to 22/39 (56 %) of the patients. **Conclusion**: In an effort to monitor pin site infections in this complex patient group and to ensure the best clinical outcomes, our registration procedure in the outpatient clinic helped to recognize pin site infections early and eased communication between team members providing a concise overview of the treatment course.

## 1 Introduction

External ring fixation is a well-established treatment modality in trauma, limb deformity, and bone infection surgery (Green, 1992; Green et al., 1992; Rajacich et al., 1992; Tucker et al., 1992; Watson, 1994b, a). However, there is a risk of acquiring pin site infections, which can occur during the entire treatment period. Pin site infection is the most frequent complication for external ring fixators with a reported incidence ranging from 10 % to 100 % depending on the indication, patient-related factors, pin site care, as well as the duration of treatment (Ferreira and Marais, 2012; Jauregui et al., 2015; Schalamon et al., 2007; Lobst, 2017; Ferguson et al., 2021). Superficial pin site infections can progress to major deep complications, e.g. severe soft tissue infection and acute and chronic osteomyelitis. Ultimately, this can result in frame abandonment with significant morbidity and mortality risk for the patient. In 1993, Checketts and Otterburn (CO) published a classification system for pin site infections to support treatment decision-making (Checketts et al., 1993) (Table 1). The CO-classification grades pin site infections into two essential groups: minor infection (CO 1–3) and major infection (CO 4–6), with involvement of bone tissue in the latter, leading to pin loosening.

**Table 1:** Checketts–Otterburn classification (Checketts et al., 1993) grades pin site infections into minor infection (CO 1–3) and major infection (CO 4–6). Neither infection nor inflammation grades as CO 0.

Grade	Characteristics	Grade	Characteristics
	Minor infection		Major infection
1	Minor infection: slight redness, little discharge	4	Major infection: severe soft tissue infection involving several pins sometimes with associated loosening of the pin
2	Minor infection: redness of the skin, discharge, pain and tenderness in the soft tissues	5	As grade 4 but also involvement of the bone, also visible on radiographs
3	As grade 2 but not improved with oral antibiotic	6	This infection occurs after fixator removal. The pin track heals initially but will subsequently break down and discharge in intervals. Radiographs shows new bone formation and sometimes sequestra.

Whereas the causes of pin site infections are adequately described in the literature, monitoring of onset, location, and the distribution of infection among the pin sites still needs further attention. In order to monitor pin site infections, facilitate communication between team members, and assist treatment decision-making, we aimed to develop a registration tool. The present pilot study evaluates the feasibility of a prospective registration procedure for reporting, evaluating, and monitoring of pin site infections in 39 trauma, limb deformity, and bone infection patients with external ring fixation.

## 2 Patients and methods

In this prospective, single-centre case series of pin site infections were monitored with a novel registration tool, which was introduced at the Department of Orthopaedic Surgery, Aarhus University Hospital, as a part of standard medical documentation in November 2017 (Supplement).

Between November 2017 and January 2019, 49 consecutive patients were treated with an external ring fixation and included in this study. A total of 10 patients were excluded due to missing data: 2 patients were postoperatively followed at another hospital, 2 paediatric patients (1 tibial fracture and 1 limb length correction) were followed in the paediatric clinic, 5 patients (1 pilon fracture, 1 distal tibial fracture, 1 open tibia fracture, 1 ankle fusion in diabetic patient, 1 proximal tibia non-union) had no pin site registration sheet due to administrative missteps, and 1 patient died 3 weeks after the operation for reasons unrelated to primary surgery.

Follow-up data were available for 39 patients (15 female, 24 males) until frame removal, with a mean age of 49 years (range: 12–88) at the time of the primary operation. In total, we report 3296 pin site observations of 568 pin sites. All pin sites were registered and named unambiguously in the following sequence: anatomic region (femur, tibia, foot), proximal metaphysis, proximal and distal diaphysis, distal metaphysis, and side (medial, lateral). Each trans-osseous wire had two pin site registrations: entry and exit point. When several wires were inserted at the same level (e.g. in the proximal tibia metaphysis), numbering of the pin sites started from the most posterior-medial site and continued anti-clockwise on the right extremity and clockwise on left extremity.

The primary underlying diagnosis was tibial fracture: 34 cases, 15 open fractures (Gustilo type I: 3 patients, type II: 4 patients, type IIIA: 4 patients, type IIB: 4 patients) consisting of 14 proximal tibial fractures (AO type 41), 12 diaphyseal fractures (AO type 42), 10 distal metaphyseal fractures (AO type 43), and 2 malleolar fractures. Four of these fractures extended in two levels: three combined fractures of type 41+42 and one fracture of 42+43). The five remaining diagnoses were two malunions, two non-unions, and one knee arthrodesis due to failed total knee arthroplasty. All patients were operated and followed by two senior orthopedic surgeons (JDR and JP).

All patients were treated with ring fixators, and no monoliteral frames were applied: Taylor spatial frame (28 frames, Smith&Nephew), Ilizarov ring fixation (10 frames, Smith&Nephew), and TrueLok Hexapod (1 frame, Orthofix). System choice of external ring fixation was at the discretion of the surgeon.

The majority of the frames consisted of three tibial rings (29 patients: 19 proximal metaphyseal and 10 distal metaphyseal). Eight patients had two tibial rings and two had four rings. The adjacent joint was temporarily spanned in 20 cases (12 foot frames and 8 the distal femur). At metaphyseal level, the external rings were predominantly fixated with four trans-osseous 1.8 mm Ilizarov olive wires (Orthofix) of stainless steel, whereas at diaphyseal level, the rings were mainly fixated using 6 mm hydroxyapatite-coated half-pins (Orthofix) per ring. The combination of wires and half-pins, as well as their orientation and number, was at the discretion of the surgeon and surgical preference. At least three to four fixation points per bone segment were applied to secure the proper stability of the frame.

### 2.1 Pin site management

Perioperatively, non-touch pin technique with intermittent drilling under irrigation was performed when inserting the pins. Every pin site was carefully inspected for skin tension and released if necessary. Pin sites were then covered with foam sponges soaked in 70 % alcohol and 0.5 % chlorhexidine solution and gently pressed to the skin with a rubber stopper. The foam sponges were removed on the first postoperative day, and all pin sites were cleaned from remaining bloodstains and covered with new, figure of 8, split gauze dressings moistened with chlorhexidine solution. Occlusive gauze dressing was rolled above the bungs, which covered the pin sites completely. The pin dressings were changed at the day of discharge and repeated once a week by a municipal home nurse. Frequent dressing changes and increased pin site care (2–3 times per week) were our first line of management upon inflammation/superficial infection (CO 1–3). If no improvement of CO 1 was achieved or further development of infection was observed, oral (CO 2) or intravenous (CO 3) antibiotics were administered. Following our local guidelines, first choice of antibiotics was peroral dicloxacillin, 1 g, four times daily, as the majority of pin sites infections are caused by *Staphylococcus aureus* (Davies et al., 2005). No culture swabs from pin sites were performed in CO 1 or in CO 2 cases. If no effect was achieved with increased pin site care and oral antibiotics, pin site specimens were collected for microbiological analysis and intravenous antibiotics were administered guided by the microbiological growth (CO 3 cases). The decision of wire/pin removal or replacement was guided by infection grade, the duration in frame, and anatomical position and at last was left to the discretion of the treating surgeon.

### 2.2 Follow-up

An individual pin site registration sheet followed the patient throughout follow-up. The standard postoperative follow-up programme included pin site inspection in the outpatient clinic at 2 and 6 weeks and was subsequently followed with 6-week intervals until removal of the external ring fixation. All unexpected visits were registered. Outpatient pin sites care was carried out by an expert nurse who systematically evaluated all pin sites according to the CO classification. A database was created from both the individual registration sheet and from the electronic medical report (MidtEPJ version 30.1.6), registering pin sites status, outpatient visits, re-operations, use of antibiotics, and radiographics.

Missing data in the registration sheet were handled as follows: if no sign of pin site infection was noted in the electronic medical report, the pin site status was registered as CO 0. If electronic notes regarding pin site infection status were missing, the CO grade was registered as a missing value.

### 2.3 Statistical analysis

Outcome measures were pin site infection (CO grade, onset, location), use of antibiotics, treatment duration, and any unplanned procedure due to pin sites complications (wire removal and/or replacement, premature frame removal, amputation etc.). Descriptive statistics were applied.

## 3 Results

In all patients, the external ring fixation had been removed at the time of the data analysis. The mean (SD) frame time was 164 (83) d (range: 44–499). Six femoral pin sites had missing CO registration. Pin infection was registered at 171 of the 568 pin sites (30 %), of which 112 (65 %) were categorized as CO 1, 42 (25 %) as CO 2, 9 (5 %) as CO 3, and 8 (5 %) as CO 5. Please refer to Table 2 for a detailed overview of anatomical pin site locations, quantity, and CO grading.

**Table 2:** Overview of anatomical pin site locations, quantity, and CO grading. Miss: missing information.

Limb segment	Pin sites	Pin site infections (CO grade)							
		CO 0	CO 1	CO 2	CO 3	CO 4	CO 5	Miss	Total
Femur (middle and distal diaphysis)	Wires	8	5	2	0	0	0	2	16
Number of rings: 9	Half-pins	6	5	5	0	0	0	4	18
	Total pin sites	14	10	7	0	0	0	6	37
Tibia (proximal metaphysis)	Wires	79	40	18	5	0	8	0	148
Number of rings: 22	Half-pins	10	5	0	0	0	0	0	15
	Total pin sites	89	45	18	5	0	8	0	165
Tibia (proximal diaphysis)	Wires	19	4	4	1	0	0	0	28
Number of rings: 40	Half-pins	60	8	3	0	0	0	0	71
	Total pin sites	79	12	7	1	0	0	0	99
Tibia (distal diaphysis)	Wires	10	5	1	0	0	0	0	16
Number of rings: 38	Half-pins	63	5	3	0	0	0	0	71
	Total pin sites	73	10	4	0	0	0	0	87
Tibia (distal metaphysis)	Wires	62	23	4	2	0	0	0	86
Number of rings: 15	Half-pins	7	3	1	0	0	0	0	9
	Total pin sites	69	26	5	2	0	0	0	102
Foot frame	Wires	66	9	1	0	0	0	0	76
Number of rings: 13	Half-pins	1	0	0	1	0	0	0	2
	Total pin sites	67	9	1	1	0	0	0	78
	Sum	391	112	42	9	0	8	6	568

A total of 35 patients (90 %) encountered a minor infection (CO 1–3) at least once during the observation time. A total of 19 of these 35 patients (54 %) were treated sufficiently with increased pin site care (n=10) or oral antibiotics (n=9). A total of 12 patients (34 %) had a wire removed in the outpatient clinic. In three of these, a wire was removed at CO 1 without previous antibiotics because it did not affect the stability of the frame. Four patients (11 %) were treated with wire removal and replacement in the operating room. One patient (2.5 %) with dysregulated diabetes mellitus developed a deep infection proximal tibia metaphysis (CO 5) that was not amenable for further limb preserving treatment and was amputated. Treatment of the pin site infections according to the highest registered CO grade is shown in Table 3.

**Table 3:** Treatment of pin sites infection according to the highest registered CO grade.

CO grade	No. of patients	Treatment					
		Increased pin	Oral	Wire removal	AB + wire	AB + wire removal	Amputation
		site care	antibiotics	no AB	removal	and replacement	
1	16	7	3	3	2	1	
2	12	3	5		3	1	
3	6				4	2	
4							
5	1						1
Missing${}^{*}$	1		1				
Total	36	10	9	3	9 (2 oral + 7 IV)	4	1

AB: antibiotics. IV: intravenous. ${}^{*}$ Missing received oral antibiotics and no wire removal, but no CO grade was reported. All patients treated with oral antibiotics, wire removal, and replacement initially received increased pin site care.

The mean (SD) time to onset of pin site infection was 34 (33) d (range: 7–149). In 10 patients, the infection started at a single pin site, and the remaining 26 infections were registered at several pin sites. The proximal tibia diaphysis was the most common anatomical location for infection start, while the foot accounted for the fewest cases.

Antibiotics were administered to 22/39 (56 %) of the patients (21 oral and 2 both intravenous and oral administration) in the postoperative period, and the mean (SD) time from surgery to start of antibiotic treatment was 40 (39) (range: 2–141) d. In total, 10/39 (26 %) patients received antibiotics for more than 2 weeks. Infection frequency (%) by anatomical location is depicted in Table 4.

**Table 4:** Infection frequency (%) by anatomical location.

Femur (middle & distal diaphysis)	45 %
Tibia (proximal metaphysis)	46 %
Tibia (proximal diaphysis)	20 %
Tibia (distal diaphysis)	16 %
Tibia (distal metaphysis)	32 %
Foot	14 %

## 4 Discussion

In this single-centre prospective evaluation of pin site infections in 39 trauma, limb deformity, and bone infection patients treated with external ring fixation, we employed a simple registration procedure combining pin site locations and CO grade, offering a feasible and easy-to-use instrument for health-care professionals to monitor pin site infections and providing a concise overview of treatment course.

Treatment with external ring fixation involves several health-care professionals forming a multidisciplinary team; therefore viable inter-team communication is of utmost importance. While several pin site infection classification systems exist (Clint et al., 2010; Patterson, 2005), the outpatient registration tool presented in the present study is combined with the validated CO-classification system (Checketts et al., 1993). This provided us with an overview of all frame pin sites and treatment course, which proved to be efficient for the workflow in the outpatient clinic. Early recognition of infection and initiation of relevant treatment is crucial to prevent major late complications, e.g. frame abandonment and severe soft tissue and bone infections. Therefore, besides providing adequate surgery, all centres treating trauma, limb deformity, and bone infection patients with external ring fixation should strive for sufficient postoperative and outpatient monitoring to ensure the best clinical outcomes.

The current inconsistent evidence of pin site infection rates is primarily defined by two key factors: (1) treatment length and (2) the infection rate vary if expressed as the number of pin sites (a trans-osseous wire has two entry points, while a half-pin has one) or the number of patients. Prudently, treatment length correlates with higher risk and incidence of infection. Of the included 568 pin sites, 171 were infected (30 %), but 36 patients (92.5 %) in this cohort developed a pin site infection according to the CO classification. Of these, 16 patients (41 %) were only graded as CO 1, in which infection was resolved in 7 of the patients with increased pin site care. In 12 of the patients (31 %), the highest CO grade registered was CO 2, in 6 of the patients (15.5 %) CO 3, in 1 patient (2.5 %) CO 5, and in 1 patient (2.5 %) no CO grade was reported. Our findings parallel the existing literature demonstrating that pin site infections are common but also that most pin site infections are categorized as minor and only few lead to major infections (Green, 1983; Ferreira and Marais, 2012; Piza et al., 2004).

Evidently, the cause of pin site infection is multifactorial. Among important factors are surgical and pin care performance, anatomical location, bacterial aetiology, and patient-related factors, e.g. comorbidity, intake of medication, nutrition status, and smoking. As illustrated by our anatomical distribution of results, pin sites near joints have a larger risk of becoming infected possibly due to movement of skin around the pin sites causing irritation of soft tissue and accumulation of fluid (Davies et al., 2005; Mahan et al., 1991; Clasper et al., 2001). Notably, we report no intraarticular infections. The most common aetiology of pin site infections is *Staphylococcus aureus* frequently responding readily to oral antibiotics if located superficially (Davies et al., 2005). The mean (SD) time to onset of pin site infection was 34 (33) d, most often starting at several pin sites, necessitating antibiotic administration to 56 % of the patients. In 15 of the infected patients (42 %), a wire was removed, which is suggestive of an insufficient effect of antibiotics and possible infection involvement of the deeper tissue layers. However, we frequently remove wires in the later stages of healing when the activity level of the patient increases and the pin site troubles the patient due to mechanical irritation. In accordance with this notion, no radiolucency was observed around the wires/pins on radiographs; therefore the pin site infection registration remained < CO 4. In terms of pin care performance, a systematic Cochrane review by Lethaby et al. (2013) assessed the effect on infection rates of different methods of cleansing and dressing orthopaedic percutaneous pin sites and found insufficient evidence to identify a strategy of pin site care that minimizes infection rates, mainly due to large clinical variations in patient status and treatment regimens (Lethaby et al., 2013). Equally, a recent prospective randomized study found no differences in a traditional versus an emollient skincare regimen (Ferguson et al., 2021). However, W-Dahl et al. found that chlorhexidine solution as a cleansing agent was superior to sodium chloride (W-Dahl and Toksvig-Larsen, 2004) and furthermore found no difference in daily and weekly pin site care (W-Dahl et al., 2003). In the present study population, the heterogeneity of the patients and indications for treatment omits the possibility of evaluating the influence of external factors. Future prospective studies assessing the individual and combined impact of external factors and aspiring for high-quality strategies for the best prevention of pin site infections are warranted.

This study has a number of limitations. The included study population represents a heterogenous but genuine unselected (i.e. consecutive) cohort, as no strict exclusion criteria were employed. The registration procedure is time-consuming and requires trained and experienced health-care professionals, both in terms of evaluating the pin sites for CO grade and filling out the registration sufficiently. In this context, we failed to include five patients due to administrative missteps, illustrating the necessity of complete inter-team communication. Moreover, evaluation of pin sites and surrounding tissue is often subjective and can differ between clinicians, which may lead to either over- or underestimation of the results (Ceroni et al., 2016). However, CO grading can be used to ensure a reliable and reproducible assessment and to collect the data for further development of treatment strategies.

In conclusion, pin site infection is a common complication with external ring fixation. During a mean frame time of 164 d, we registered infection in 30 % of the pin sites but in 92.5 % of the patients according to the CO classification. In an effort to monitor all pin site infections in this complex patient group and to ensure the best clinical outcomes, our registration procedure in combination with the CO classification helped early diagnosis of pin site infections and eased communication and workflow in the outpatient clinic by providing a concise overview of treatment course. Future studies should include further understanding and optimization of of post-operative pin site care protocol and prospectively evaluate the influence of external factors on the development of pin site infections.

## Supplement

10.5194/jbji-6-135-2021-supplement The supplement related to this article is available online at: https://doi.org/10.5194/jbji-6-135-2021-supplement.

## Data Availability

The data that support the findings of this study are available from the corresponding author upon reasonable request.

## References

[bib1.bib1] Ceroni D, Grumetz C, Desvachez O, Pusateri S, Dunand P, Samara E (2016). From prevention of pin-tract infection to treatment of osteomyelitis during paediatric external fixation. J Child Orthop.

[bib1.bib2] Checketts RG, MacEachern AG, Otterburn M (1993). Pin track infection: definition, incidence and prevention. Int J Orthop Trauma Suppl.

[bib1.bib3] Clasper JC, Cannon LB, Stapley SA, Taylor VM, Watkins PE (2001). Fluid accumulation and the rapid spread of bacteria in the pathogenesis of external fixator pin track infection. Injury.

[bib1.bib4] Clint SA, Eastwood DM, Chasseaud M, Calder PR, Marsh DR (2010). The “Good Bad and Ugly” pin site grading system: A reliable and memorable method for documenting and monitoring ring fixator pin sites. Injury.

[bib1.bib5] Davies R, Holt N, Nayagam S (2005). The care of pin sites with external fixation. J Bone Joint Surg Br.

[bib1.bib6] Ferguson D, Harwood P, Allgar V, Roy A, Foster P, Taylor M, Moulder E, Sharma H (2021). The PINS Trial: a prospective randomized clinical trial comparing a traditional versus an emollient skincare regimen for the care of pin-sites in patients with circular frames. Bone Joint J.

[bib1.bib7] Ferreira N, Marais LC (2012). Prevention and management of external fixator pin track sepsis. Strategies Trauma Limb Reconstr.

[bib1.bib8] Green SA (1983). Complications of external skeletal fixation. Clin Orthop Relat R.

[bib1.bib9] Green SA (1992). Ilizarov method. Clin Orthop Relat R.

[bib1.bib10] Green SA, Jackson JM, Wall DM, Marinow H, Ishkanian J (1992). Management of segmental defects by the Ilizarov intercalary bone transport method. Clin Orthop Relat R.

[bib1.bib11] Jauregui JJ, Bor N, Thakral R, Standard SC, Paley D, Herzenberg JE (2015). Life- and limb-threatening infections following the use of an external fixator. Bone Joint J.

[bib1.bib12] Lethaby A, Temple J, Santy-Tomlinson J (2013). Pin site care for preventing infections associated with external bone fixators and pins. Cochrane Database Syst Rev.

[bib1.bib13] Lobst C (2017). Pin-track infection: past, present and future. J Limb Lengthen Reconstr.

[bib1.bib14] Mahan J, Seligson D, Henry SL, Hynes P, Dobbins J (1991). Factors in pin tract infections. Orthopedics.

[bib1.bib15] Patterson MM (2005). Multicenter pin care study. Orthop Nurs.

[bib1.bib16] Piza G, Caja VL, Gonzalez-Viejo MA, Navarro A (2004). Hydroxyapatite-coated external-fixation pins. The effect on pin loosening and pin-track infection in leg lengthening for short stature. J Bone Joint Surg Br.

[bib1.bib17] Rajacich N, Bell DF, Armstrong PF (1992). Pediatric applications of the Ilizarov method. Clin Orthop Relat R.

[bib1.bib18] Schalamon J, Petnehazy T, Ainoedhofer H, Zwick EB, Singer G, Hoellwarth ME (2007). Pin tract infection with external fixation of pediatric fractures. J Pediatr Surg.

[bib1.bib19] Tucker HL, Kendra JC, Kinnebrew TE (1992). Management of unstable open and closed tibial fractures using the Ilizarov method. Clin Orthop Relat R.

[bib1.bib20] Watson JT (1994). Treatment of unstable fractures of the shaft of the tibia. J Bone Joint Surg Am.

[bib1.bib21] Watson JT (1994). High-energy fractures of the tibial plateau. Orthop Clin North Am.

[bib1.bib22] W-Dahl A, Toksvig-Larsen S (2004). Pin site care in external fixation sodium chloride or chlorhexidine solution as a cleansing agent. Arch Orthop Trauma Surg.

[bib1.bib23] W-Dahl A, Toksvig-Larsen S, Lindstrand A (2003). No difference between daily and weekly pin site care: a randomized study of 50 patients with external fixation. Acta Orthop Scand.

