# Supplementary material for: Prospective evaluation of pin site infections in 39 patients treated with external ring fixation
Source: J Bone Jt Infect. 2021 Apr 7;6(5):135–40. doi: 10.5194/jbji-6-135-2021 (PMC8131963; doi:10.5194/jbji-6-135-2021)

Supplement of J. Bone Joint Infect., 6, 135–140, 2021
https://doi.org/10.5194/jbji-6-135-2021-supplement
© Author(s) 2021. CC BY 4.0 License.

JBJI
Journal of Bone and Joint Infection
Open Access

Supplement of

Prospective evaluation of pin site infections in 39 patients treated with external ring fixation

Mats Bue et al.

Correspondence to: Juozas Petruskevicius (juopet@rm.dk)

The copyright of individual parts of the supplement might differ from the article licence.

Supplement
Pin site registration sheet.

Table S1:
Name of the patient + ID: ___________________________________
Date of primary ring fixation (dd-mm-yy): _________________

Follow-up

	Date dd/mm-yy	**Antibiotics since last visit** (Yes/No)	**Comments** (Pin care frequency,Antibiotics, dosage, hospitalisation, other events)
1			
2			

Name of the patient + ID: ___________________________________
Operations
(primary ring fixation, re-operations, wire/pin removal, removal of rings, etc)

	Date dd/mm-yy	**Comments**
1		
2		

Figure S1:
Draw the frame on the actual limb and side.

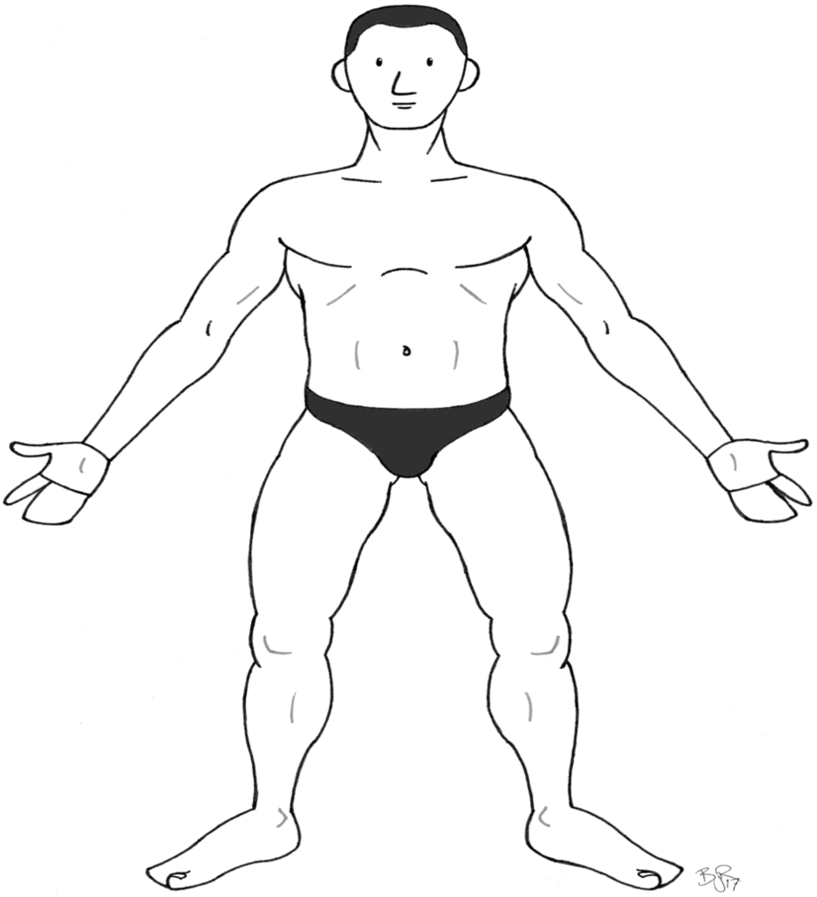

Anatomical location
Definition of the rings:

Femur: **D**istal **d**iaphysis, (F-DD)
Distal **m**etaphysis (F-DM)

Tibia: **P**roximal **m**etaphysis (PM)
Prox **d**iaphysis (PD)
Distal **d**iaphysis (DD)
Distal **m**etaphysis (DM)

Foot: Foot frame

Table S2:

Halfpins (HP) and Wires

- Numbering of the pin sites starts from the most posterior-medial site and continues anti-clockwise on the right extremity (clockwise on left extremity): HP1, HP2 for halfpins, 1M, 2M for wires and so on)
- A wire always has two pin sites: medial and lateral (ex: 2M and 2L).

Registration of pin site infection
Note the grade of infection (CO 0-6, see at bottom of this sheet) in the empty cells

Date	**Tibia – Prox metaphysis**									Comments
	1M	2M	3M	4M	1L	2L	3L	4L		

Date	**Tibia – Prox diaphysis**				Comments
	HP1	HP2			

Date	**Tibia – Distal diaphysis**				Comments
	HP1	HP2			

Date	**Tibia – Distal metaphysis**									Comments
	1M	2M	3M	4M	1L	2L	3L	4L		

Date	**Femur – Distal diaphysis**					Comments
	HP1	HP2	1M	1L		

Date	**Femur – Distal metaphysis**					Comments
	HP1	HP2	1M	1L		

Date	**Footframe**							Comments
	1M	2M	3M	3L	1L	2L		

Figure 2S:
Femur: distal diaphysis or distal metaphysis with wires and half pins

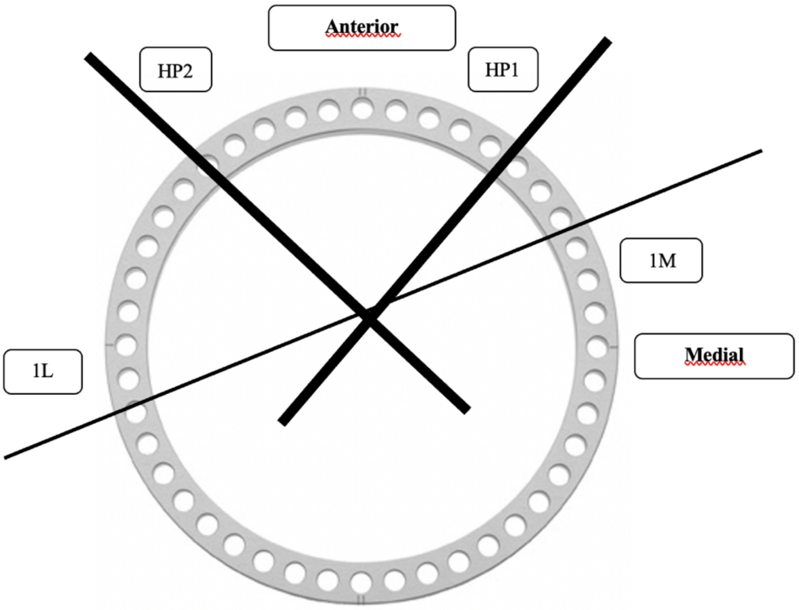

Figure 3S:
Tibia: proximal/distal metaphysis with wires (standard placement)

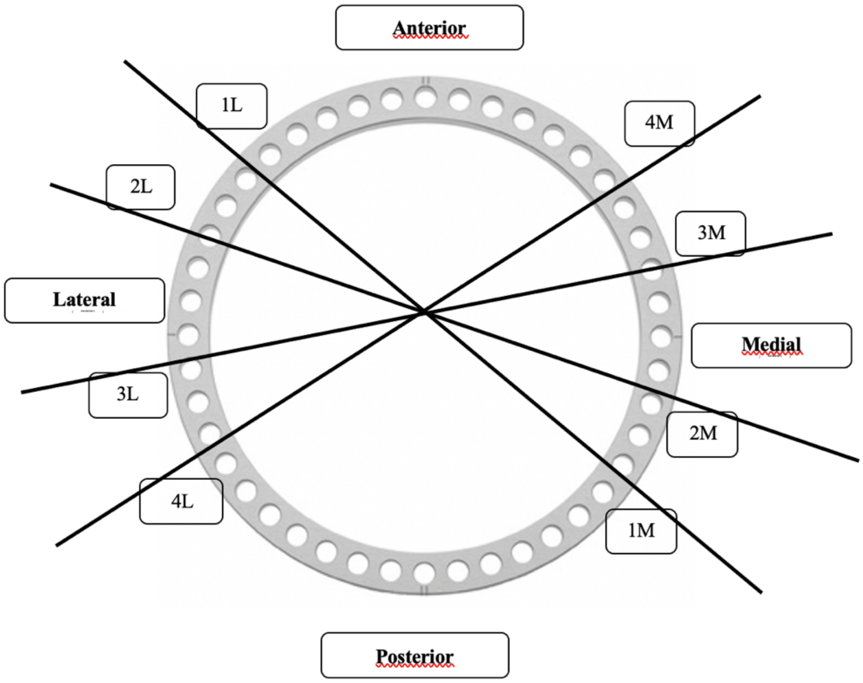

Figure 4S:
Tibia: proximal or distal diaphysis with half pins (standard placement)

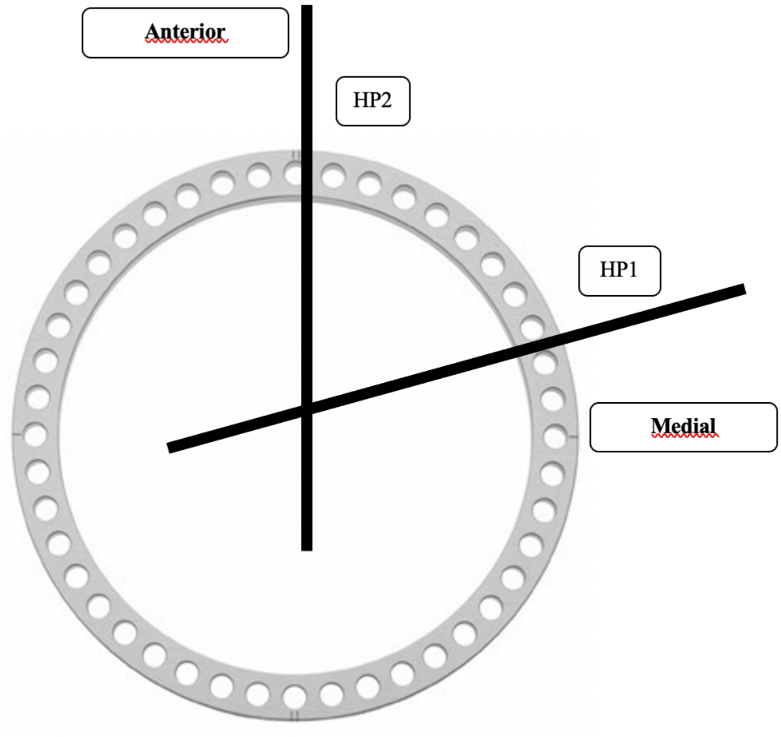

Figure 5S:
Footframe: pin sites numbering for standard placement for wires og half pin.

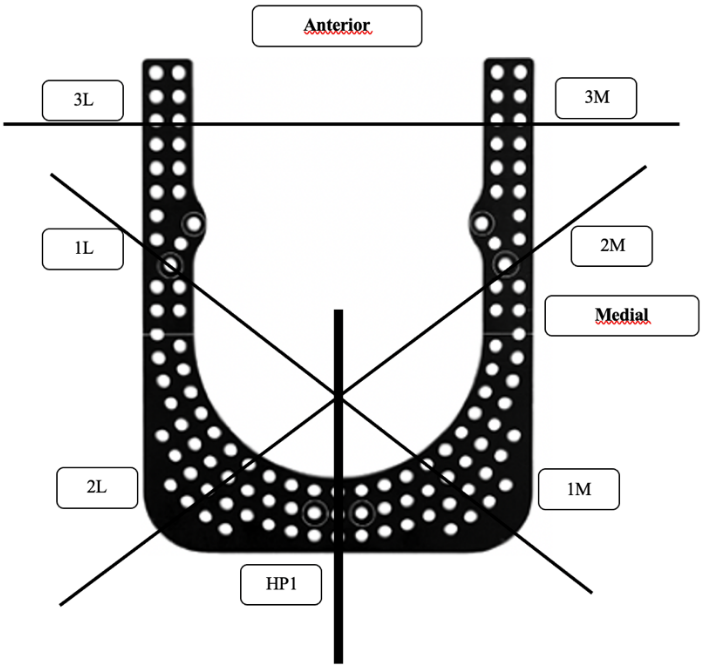

Supplement: The supplement related to this article is available online at: https://doi.org/10.5194/jbji-6-135-2021-supplement. [file jbji-6-135-supplement.pdf]
